# Surrogate Decision-Making in Carceral Healthcare

**DOI:** 10.1007/s11606-026-10393-8

**Published:** 2026-04-20

**Authors:** Anurima Chattopadhyay, Daniel Karel, David Wendler

**Affiliations:** Department of Bioethics, National Institutes of Health, Bethesda, MD USA

**Keywords:** surrogate decision-making, carceral healthcare, conflicts of interest

## Abstract

The carceral healthcare system is reckoning with a rapidly aging patient population. This is significant because elderly patients are more likely to have medical conditions that render them unable to make their own decisions. Existing laws and ethical guidelines mandate relying on patient-designated and next-of-kin surrogates to make medical decisions for patients who lack decision-making capacity. Yet, providing healthcare to incarcerated patients who lack decisional capacity is especially challenging. As a result, practices vary, and correctional officials, such as wardens or guards, are frequently involved in making medical decisions for incarcerated patients who lack decision-making capacity. The present paper thus considers what qualities make for effective surrogate decision-makers and whether correctional officials tend to possess them. We conclude that correctional officials frequently lack these qualities before exploring other approaches to surrogate decision-making. We identify several approaches with greater potential to ensure that incarcerated individuals’ treatment is consistent with their preferences and values.

## SURROGATE DECISION-MAKING IN CARCERAL HEALTHCARE

Approximately 180,000 of the 1.2 million individuals incarcerated in US state and federal prisons are over the age of 55, a proportion five times larger than three decades ago^1,2^. This increase poses significant challenges because elderly patients are more likely to have medical conditions that render them unable to make decisions. In one study, nearly 50% of hospitalized patients aged 60 years or older needed a surrogate decision-maker^3^.

Laws in many states mandate relying on patient-designated and next-of-kin surrogates to make medical decisions for patients who lack decision-making capacity, whether incarcerated or not^4–6^. Consistent with this mandate, the US Bureau of Prisons prohibits correctional officials from acting as surrogates in federal prisons^7^. Yet, correctional officials are involved in surrogate decision-making for many incarcerated patients in other prisons. While there are limited data on how often this occurs, a study at a major academic medical center found that correctional officials were involved in making medical decisions for incarcerated patients in approximately 50% of cases and served as the *primary*surrogate in about one-third of these cases^8^.

In response, we assess what qualities surrogate decision-makers should have, concluding that correctional officials frequently lack these qualities. We then consider approaches which have greater potential to ensure incarcerated individuals’ treatment is consistent with their preferences and values regarding who makes treatment decisions for them and which treatments they receive.

## CURRENT PRACTICE

As of 2022, 44 states have surrogate decision-making laws. Most of these laws mandate relying on patient-designated and next-of-kin surrogates^4^. In addition, 12 states explicitly prohibit correctional officials from serving as surrogates for incarcerated patients who cannot make their own decisions^9^. Federal prisons maintain a similar prohibition^7^. Nevertheless, correctional officials are frequently involved in surrogate decision-making^8^.

This practice likely traces to several factors. First, incarcerated individuals rarely designate a surrogate for themselves: fewer than 1% have an advance directive, compared to the approximately 36% of non-incarcerated patients who do^10,11^. This difference may be due, in part, to the fact that regulations regarding advance directives vary widely across carceral institutions, with some facilities prohibiting incarcerated individuals from completing them^12–14^.

Many incarcerated individuals are also estranged from their families, leaving them with limited options for designating a surrogate^15^. Estrangement can also make it challenging for clinicians to locate a next-of-kin surrogate. Moreover, incarcerated patients’ designated or next-of-kin surrogates frequently face significant obstacles traveling to carceral facilities or hospitals^16^, and some correctional officials may prevent family from visiting the patient^16,17^. Although surrogates *can*make decisions over the phone, in-person decision-making is preferable^18^. Institutions and clinicians may thus regard correctional officials as the only available decision-maker.

Even when an incarcerated patient’s designated or next-of-kin surrogate is available, correctional officials may still be involved. Correctional officials are necessarily in close proximity when healthcare is delivered in carceral facilities. When healthcare is delivered outside carceral facilities, correctional officials typically accompany patients. Since correctional officials have authority over incarcerated individuals, clinicians may feel obligated to defer to them, despite no legal or ethical requirement to do so. In these cases, correctional officials’ involvement in surrogate decision-making may involve co-consent, with clinicians asking correctional officials to “sign off” on decisions, potentially enabling them to override decisions made by the patient’s designated or next-of-kin surrogate^8^.

## APPROPRIATE SURROGATES

To protect and respect patients who lack decisional capacity, surrogates are instructed to make decisions based on a widely accepted hierarchy: (1) adhere to the patient’s prior directives, if any; (2) make the decisions the patient would have made (i.e., utilize substituted judgement); and (3) promote the patient’s best interests^19^. What qualities help surrogates to promote these goals?

First, surrogates literally make life-and-death decisions; hence, it is important that they care for the patient and are committed to respecting their preferences and values. Second, to effectively implement substituted judgement, surrogates should know the patient. Ideally, they should have a sense for the treatments the patient would want or, more generally, the type of life the patient endorsed for themselves. Third, while all individuals have *some* conflicts of interest, it is important that surrogates have minimal fundamental conflicts that could undermine promotion of the three goals.

Fourth, to make medical decisions, surrogates need to know patients’ medical information. Their surrogate should thus be someone the patient would be comfortable knowing this information. Fifth, and finally, a patient’s surrogate should be someone the patient would want to make decisions for them.

## RELYING ON CORRECTIONAL OFFICIALS

Correctional officials are charged with carrying out incarcerated individuals’ punishment. As a result, they necessarily have a coercive relationship with incarcerated individuals. Given this power dynamic, correctional officials and incarcerated individuals rarely have the type of caring relationship that is critical for surrogate decision-making; they may even have antagonistic relationships.

Correctional officials’ relationships to incarcerated individuals are also typically limited to the duration of the individual’s incarceration. As a result, they are unlikely to be aware of the type of life the individual led and endorsed for themselves.

Even correctional officials who care about an incarcerated patient have “dual loyalty”—professional obligations that can conflict with their role as surrogate^20^. This can introduce fundamental conflicts of interest. For example, to avoid their facility facing litigation for not providing adequate medical care, correctional officials may opt for aggressive treatment, even when the patient would not want or need it. Conversely, given that carceral facilities often bear the cost of incarcerated patients’ healthcare, correctional officials may opt against expensive treatments to protect their facility’s financial interests.

Incarcerated individuals already have limited privacy in carceral facilities. Hence, many likely prefer that correctional officials are not informed of their private medical information during the surrogate decision-making process^21^. Lastly, incarcerated individuals may not want correctional officials to act as their surrogate. These five considerations, taken together, suggest that correctional officials frequently do not have the qualities that make for effective surrogates, thus highlighting the need to pursue approaches that do not include correctional officials in surrogate decision-making for incarcerated patients.

## ALTERNATIVE APPROACHES

Due to mistrust of correctional officials and healthcare staff, many incarcerated individuals do not complete an advance directive^22^. Correctional healthcare staff should thus seek to build trusting relationships with their patients, discuss patients’ treatment goals, and encourage them to complete an advance directive (Table 1). Historically, there has been a lack of training for clinicians on how to best provide healthcare to incarcerated patients. In response, there is a need for more training, especially with respect to surrogate decision-making^8,13,23^.

**Table 1:** Best Practices for Surrogate Decision-Making for Incarcerated Patients

Reduce barriers (e.g. travel, security, and communication) to facilitate patient-designated or next-of-kin surrogates making medical decisions
Increase clinician education about legal and ethical practices when caring for incarcerated patients who are being treated in carceral settings or community hospitals
Refrain from seeking co-consent from correctional officials when a patient-designated or next-of-kin surrogate exists
Revise carceral policies preventing incarcerated individuals from completing advance directives; increase the rates of advance medical planning among incarcerated individuals
For unrepresented patients treated outside carceral medical facilities, defer to existing guidelines and laws on surrogate decision-making for non-incarcerated, unrepresented patients
For unrepresented patients treated within carceral medical facilities, utilize local clinicians or local hospitals’ ethics committees to make decisions
For surrogate decision-making in general, consider supplementing surrogates’ judgment with input from prison family or a patient preference predictor if it becomes available

Compared to correctional officials, patient-designated and next-of-kin surrogates are more likely to have caring relationships with the patient, know the patient’s wishes, and be someone the patient is comfortable with knowing their private medical information and making medical decisions for them. Hence, clinicians should be trained to prioritize communicating with the patient’s designated or next-of-kin surrogate before seeking alternative decision-makers. This should include proactively considering steps their institutions might take to reduce travel barriers and to address any security concerns raised by involving the patient’s surrogate. When obtaining consent from the patient’s surrogate, clinicians should also refrain from seeking co-consent from correctional officials.

Despite these efforts, it is likely that many incarcerated patients will not have a designated or available next-of-kin surrogate. Who should make decisions for these “unrepresented” patients (Table 2)? For patients treated *outside*prison walls, many hospitals have guidelines regarding medical decision-making for unrepresented patients. Some rely on institutional officials, such as physicians. Some declare the patient a ward of the state and seek a court-appointed surrogate, such as a social worker or lawyer^24^. Still others rely on their ethics committee, which usually includes clinicians, social workers, bioethicists, and community members^25,26^. These hospitals should follow the same guidelines when treating unrepresented, incarcerated patients.

**Table 2:** Evaluation of Alternative Surrogate Decision-Makers for Unrepresented Incarcerated Patients

	Caring relationship with patient?	Likely to know patient wishes?	No fundamental conflicts of interest?	Acceptable to know patient’s information?	Surrogate acceptable to patient?
Patient-designated or next-of-kin surrogate	✓	?	✓	✓	✓
Correctional official	X (antagonistic)	X	X	X	?
Court-appointed surrogate/ethics committee	X	X	✓	?	?

This leaves the challenge of making decisions for unpresented patients who are treated *within*prison walls. One option is to adopt similar approaches to those used by hospitals, such as relying on institutional physicians. However, relying on correctional physicians raises similar worries about fundamental conflicts of interest that arise when relying on correctional officials. While utilizing court-appointed surrogates would avoid these fundamental conflicts of interest, the public guardianship system is overburdened, and court-appointed surrogates may not be able to devote adequate time and care to this responsibility^27^. Additionally, like correctional officials, these surrogate decision-makers may opt for patients to receive all available life-sustaining treatment in order to avoid potential legal liability, independent of the patient’s preferences^28^.

A better approach may be for carceral institutions to rely on local community clinicians or ethics committees who have experience making decisions for unrepresented patients. Ethics committees especially, in virtue of including people with a range of backgrounds, are more likely to incorporate diverse viewpoints and better represent the patient’s interests, both medical and non-medical^25^.

More generally, the surrogate decision-making process faces significant challenges. Even patient-designated and next-of-kin surrogates are frequently unable to accurately predict patients’ treatment preferences^29^. And, up to one in three surrogates experiences symptoms associated with PTSD as a result of making treatment decisions for incapacitated patients^30^. This concern may be particularly relevant for correctional officials who are saddled with the burdens of making medical decisions for many patients whose treatment preferences they do not know. Given these concerns, it is important to consider ways to increase the chances that incapacitated patients are treated consistent with their preferences and reduce the psychological burden of making decisions on their surrogates.

Local clinicians or ethics committees could be given an opportunity to solicit information about the patient’s treatment preferences from members of their “prison family,” other incarcerated individuals who the patient became close to while incarcerated^16,31^. If clinicians adopt this proposal, they should take steps to ensure the individuals are actually close to the patient.

Relatedly, the Federal Bureau of Prisons stipulates that incarcerated individuals should not serve as surrogates for incarcerated patients^7^. However, incarcerated patients may prefer that a member of their prison family, who may know them best, act as their surrogate. Hence, absent compelling reasons not to, incarcerated patients should be permitted to designate other incarcerated individuals as their surrogates.

Some commentators have proposed to supplement surrogate decision-making with a patient preference predictor (PPP). The PPP is a proposed algorithm that would provide surrogates with a prediction of the patient’s treatment preferences based on their sociodemographics, medical records, and other information about them^32^. While the PPP should not replace surrogates, it could be used as a resource for information about the patient’s treatment preferences. This approach may increase the chances that patients who lack decisional capacity are treated consistently with their treatment preferences and values. To that end, if a PPP does become available, it will be important to train it on treatment preference data of incarcerated individuals specifically. Clinicians should also remain cognizant that systemic bias may affect the information that is included in a PPP. For example, the medical records of Black patients, who are incarcerated at disproportionately higher rates, are 2.5 times more likely to contain negative descriptors compared to White patients^33,34^.

## CONCLUSION

The surrogate decision-making process for both incarcerated and non-incarcerated patients raises significant concerns and needs improvement. Still, incarcerated patients deserve a process that is as good as the process used for non-incarcerated patients. Their surrogates should care about them, know their wishes, have minimal conflicts of interest, be good stewards of private medical information, and be acceptable to the patient. Clinicians should make every effort to contact the patient’s designated or next-of-kin surrogate. To ensure that the treatment of incarcerated individuals is consistent with their preferences and values, it will be important to pursue approaches to surrogate decision-making for unrepresented, incarcerated patients that do not rely on correctional officials.

## Declarations

### Disclaimer

The contributions of the NIH authors were made as part of their official duties as NIH federal employees, are in compliance with agency policy requirements, and are considered Works of the United States Government. However, the findings and conclusions presented in this paper are those of the authors and do not necessarily reflect the views of the NIH or the U.S. Department of Health and Human Services.
